# Explainable Fall Risk Prediction in Older Adults Using Gait and Geriatric Assessments

**DOI:** 10.3389/fdgth.2022.869812

**Published:** 2022-05-06

**Authors:** Anup Kumar Mishra, Marjorie Skubic, Laurel A. Despins, Mihail Popescu, James Keller, Marilyn Rantz, Carmen Abbott, Moein Enayati, Shradha Shalini, Steve Miller

**Affiliations:** ^1^Department of Electrical Engineering and Computer Science, University of Missouri, Columbia, MO, United States; ^2^Sinclair School of Nursing, University of Missouri, Columbia, MO, United States; ^3^Department of Health Management and Informatics, University of Missouri, Columbia, MO, United States; ^4^School of Health Professions, Physical Therapy, University of Missouri, Columbia, MO, United States; ^5^Department of Health Sciences Research, Mayo Clinic, Rochester, MN, United States; ^6^Department of Gastroenterology and Hepatology, Mayo Clinic, Rochester, MN, United States

**Keywords:** fall risk, older adults, explainable AI, geriatric assessments, gait, GAITRite, fall prediction, machine learning (ML)

## Abstract

Older adults aged 65 and above are at higher risk of falls. Predicting fall risk early can provide caregivers time to provide interventions, which could reduce the risk, potentially avoiding a possible fall. In this paper, we present an analysis of 6-month fall risk prediction in older adults using geriatric assessments, GAITRite measurements, and fall history. The geriatric assessments included were Activities of Daily Living (ADL), Instrumental Activities of Daily Living (IADL), Mini-Mental State Examination (MMSE), Geriatric Depression Scale (GDS), and Short Form 12 (SF12). These geriatric assessments are collected by staff nurses regularly in senior care facilities. From the GAITRite assessments on the residents, we included the Functional Ambulatory Profile (FAP) scores and gait speed to predict fall risk. We used the SHAP (SHapley Additive exPlanations) approach to explain our model predictions to understand which predictor variables contributed to increase or decrease the fall risk for an individual prediction. In case of a high fall risk prediction, predictor variables that contributed the most to elevate the risk could be further examined by the health providers for more personalized health interventions. We used the geriatric assessments, GAITRite measurements, and fall history data collected from 92 older adult residents (age = 86.2 ± 6.4, female = 57) to train machine learning models to predict 6-month fall risk. Our models predicted a 6-month fall with an AUC of 0.80 (95% CI of 0.76–0.85), sensitivity of 0.82 (95% CI of 0.74–0.89), specificity of 0.72 (95% CI of 0.67–0.76), F1 score of 0.76 (95% CI of 0.72–0.79), and accuracy of 0.75 (95% CI of 0.72–0.79). These results show that our early fall risk prediction method performs well in identifying residents who are at higher fall risk, which offers care providers and family members valuable time to perform preventive actions.

## Introduction

The number of Americans ages 65 and older is projected to be over 98 million by 2060, which is about 24 percent of the total population in the USA (1). Studies show that more than one-third of older adults fall each year (2). Out of these fallers, 20–30% of the individuals suffer moderate to severe injuries, which reduces independence and mobility, and increases the risk of premature death (3). Identifying older adults who are at higher risk of falls requiring interventions is challenging for clinicians (4).

Falls in older adults are multi-factorial (5). Consequently, several fall risk assessment tools have been developed and validated (5). Lusardi et al. presented a systematic review and meta-analysis analyzing fall risk assessment tools (5). In their analysis, they included several self-report measures such as the Geriatric Depression Scale (GDS), Medical Outcomes Study Short Form (SF-36), and Mini-Mental State Examination (MMSE). Also, the study included medical history questions such as a history of previous falls and requiring any ADL assistance. The analysis shows that no single test/measure demonstrates a strong post-test probability in predicting fall. Deandrea et al. performed another systematic review to provide a comprehensive list of evidence-based risk factors for falls (6). This analysis did not include SF-12 measures as a risk factor. Oshiro et al. used the predictors chosen by Deandrea et al. from the Electronic Health Records (EHR) based on psychological and medical factors, medication use, and mobility or sensory factors to predict fall risk (7). Results show that their final model had a positive predictive value of 8%, a negative predictive value of 98%, and an area under the curve (AUC) of 0.74, with a sensitivity of 67% and specificity of 68%. One issue with these analyses is they use a crisp boundary in the range of scores for each assessment instead of using the entire distribution of an assessment to predict fall risk. A common analysis overlap in these two studies suggests that medical history questions, self-reported measures, performance, and mobility-based measures are some of the most commonly used predictors to estimate fall risk in the literature. Therefore, in this study we estimated fall risk based on predictors from these three categories.

Previous studies show that machine learning can be used to predict fall risk in older adults (8–11). These studies have used different combinations of predictors including demographics, gait, and balance information, fall history, and other information from the EHR to develop machine learning models to predict future fall risk. A subset of these studies has explored different methods to find the feature importance of the predictor variables to understand the top predictors of falls in their analysis (9–11). However, none of these articles have provided explanations for individual fall risk predictions for an older adult. The feature importance information could provide an overview of which predictors are important. However, features that are not necessarily important for the entire population could possibly increase the fall risk in an individual older adult. Providing information about which predictors have increased or decreased the fall risk for an individual could provide valuable insights for personalized interventions. In this study, we address this issue by using SHAP explanations for individual predictions. Similar to the previous studies, our analysis also provides the feature importance information for the predictors in the model. Moreover, these studies have only considered gait and activity-related predictor variables for fall prediction. In contrast, we have not only considered gait and activity-related parameters but also cognitive and depression scales for fall prediction.

In this study, we develop a data-driven fall risk prediction model using demographics data, several different geriatric assessments, gait measurements, and fall history. The geriatric assessments include Activities of Daily Living (ADL), Instrumental Activities of Daily Living (IADL), Mini-Mental State Examination (MMSE), Geriatric Depression Scale (GDS), and Short Form 12 (SF12) (12–16). In addition to the different geriatric assessments, we used gait parameters to predict fall risk. Gait characteristics have been used as fall risk indicators (5). We used gait data including gait speed and Functional Ambulation Performance (FAP) scores measured using the GAITRite walkway (CIR System Inc; Clifton, New Jersey), a gold standard system for measuring spatiotemporal parameters (17). FAP score captures the gait capacity of an individual using a specific set of STPS (18, 19). FAP has been validated in several independent studies (19). We have also included fall history in the last 6 months of the older adults as a predictor.

We experimented with several supervised machine learning techniques to predict future falls including logistic regression, k-nearest neighbors, decision trees, linear SVM, and random forests. We compared their performances to find the best model that could predict future falls with high sensitivity and specificity.

In addition to constructing a model to predict fall risk, we also used explainable AI techniques, specifically SHAP (SHapley Additive exPlanations) to explain our models and the specific predictions made by the models (20). SHAP uses game theory to determine the individual contributions of the input features in predicting the outcome by a machine learning model. Lundberg et al. proposed SHAP values as a unified measure of feature importance. SHAP values attribute to each feature the change in the expected model prediction when conditioning on that feature. Considering the model has a base expected value that would be predicted if we did not know any features, SHAP values explain how to get from the base value to the current output. The additive SHAP values for the individual features will either be positive or negative, hence increasing or decreasing the model prediction value starting from the expected base prediction value. SHAP can be used to provide a global explanation of a model by describing how the individual features have an overall effect on the model's predictions. SHAP can also be used to explain a particular model prediction, for example, fall prediction for an older adult using the model by providing feature importance of the individual features for that prediction. These feature importance values otherwise known as SHAP values explain a model's prediction by suggesting which features had a larger contribution in that particular prediction. The fall risk model developed in this study depends on several aspects of functional health and mobility. An explanation of the fall risk predictions of individual older adults, providing more information about the predictors that had a higher contribution in increasing or decreasing the fall risk, provides critical clinical information for targeted interventions.

In addition to the novel methodology of explaining the individual model predictions, this analysis is filling an important gap in the literature by analyzing the data of a subgroup of the older adult population with age over-65 and a mean age of 85. According to the US Census, while those over 85 make up a small percentage of the population, this percentage has increased since 2010 (21). Previous studies have included older adult populations with their mean age ranging between 65 and 75 (8–11). Whereas, the subjects in our study were independent living older adults in an Aging in Place Facility, and the fact that the average age of the population was well into the 80's, where their age itself has a greater fall risk when compared to a younger population (22). The mean age of our older adult population was 85. In this subgroup of the over-65 population, there is not much information regarding fall risk prediction using machine learning in the literature.

In this article, we present an analysis of fall risk prediction using geriatric assessments, gait parameters, and fall history. We hypothesize that the assessment scores and fall history can be used to predict fall risk with good reliability and validity. We include a description of the methods along with experiments and results.

## Materials and Methods

### Data

We used a set of standardized geriatric assessment scores, including ADL (Short Form ADL, RAI MDS 2.0), IADL (Lawton), GDS, MMSE, and SF12 (12–16). ADL are defined as activities that are essential for independent living (12). IADL require a higher level of personal autonomy, referring to tasks that require enough capacity to make decisions through greater interaction with the environment (12). MMSE is a widely used test to evaluate the cognitive aspects of mental function (13). MMSE excludes questions concerning abnormal mental experiences and mood. GDS is a screening tool for measuring depression in older adults (14). SF-12 is a multipurpose short form that provides a generic measure of health status (15, 16). SF-12 has a mental (MCS) and a physical (PCS) component. All these assessments have good reliability and validity, and they represent different factors of health and wellbeing. Higher ADL scores indicate more ADL impairment, lower IADL scores show low function, lower MMSE scores show more cognitive impairment, higher GDS scores indicate depression, and low scores of SF-12 components indicate a low level of mental or physical health. In addition to geriatric assessments, we used gait parameters such as gait speed and FAP measured using the GAITRite walkway system (17). GAITRite walkway provides several spatiotemporal gait measurements. However, performing a pair-wise correlation analysis we found that most gait parameters were highly correlated (Pearson Correlation > 0.8) with either the FAP score or gait speed. Therefore, we chose to include FAP and gait speed in our study as the only two gait parameters. A lower FAP score indicates poorer gait ability, and lower Gait Speeds indicate poorer gait ability. Finally, we also included fall history as a predictor variable with three possible values 0, 1, and 2 for having no falls, one fall, or more than one fall in the past 6 months, respectively. Fall history should not be confused with the fall outcome. Fall outcome is a binary variable with values 0 to represent no future falls and 1 to represent future falls in the next 6 months.

Data used in this analysis were collected at TigerPlace, an Aging-in-Place facility in Columbia, MO (23, 24). TigerPlace was built as an innovative independent living environment where residents can truly age in place and never fear being moved to a traditional nursing home unless they choose to do so (23). The TigerPlace residents have access to personalized care with preventative and early illness recognition assistance through the registered nurse care coordination services and in-home sensor technologies. The geriatric assessments used in this study were obtained by the nursing staff working at TigerPlace in collaboration with the Sinclair Nursing School at the University of Missouri, Columbia. All assessments were collected at an interval of ~6 months and stored in the EHR.

In this retrospective analysis, we used the assessment data previously recorded in the TigerPlace EHR database. A total of 125 residents had both geriatric assessments and GAITRite data, from which only 98 residents were 65 and older and without any missing data. Out of the 98 older adult residents, 6 more older adults were excluded from the analysis because of data collection errors in their assessment and gait data. Therefore, our final dataset consisted of data from 92 older adult residents (57 females, 35 males, age = 86.2 ± 6.4). Only the first set of assessments available for each resident was included to avoid repeated correlated measures from the same resident. Fall events reported by nursing and facility staff were used to develop the 6-month fall outcome and fall history predictor data. Table 1 shows the demographic characteristics of the older adult participants. Table 2 shows a summary of the characteristics of the predictor variables. Table 3 shows the fall history of the participants. This study received Institutional Review Board approval at the University of Missouri, Columbia.

**Table 1:** Demographic data characteristics.

**Variable**	**Non-fallers (*n* = 61)**	**Fallers (*n* = 31)**
	**Mean (Std)/counts**	**Mean (Std)/counts**
Age	85.87 (6.19)	86.90 (6.93)
Gender	Female = 36, Male = 25	Female = 21, Male = 10

**Table 2:** Predictor data characteristics.

Variable^*^	**Non-fallers (*n* = 61)**	**Fallers (*n* = 31)**
	**Mean (Std)**	**Mean (Std)**
ADL (0–16)	0.7 (1.37)	2.0 (1.81)
IADL (0–8)	4.6 (1.35)	3.52 (1.06)
MMSE (0–30)	25.61 (4.56)	26.48 (3.84)
GDS (0–15)	2.82 (2.57)	2.35 (2.37)
SF12 - PCS (0–100)	45.26 (10.82)	36.14 (10.03)
SF12 - MCS (0–100)	52.05 (9.11)	56.13 (6.43)
FAP (40–100)	78.31 (16.0)	64.32 (15.78)
Gait speed	75.83 (25.30)	53.41 (24.92)

* *Interpretation of the variables—ADL, higher scores indicate more ADL impairment; IADL, lower scores show low function; MMSE, lower scores show more cognitive impairment; GDS, higher scores indicate depression; SF-12, low scores indicate a low level of mental or physical health; FAP, lower scores indicate poorer gait ability; Gait Speed, lower scores indicate poorer gait ability*.

**Table 3:** Fall history of study participants.

**Fall - category**	**Past falls = 0**	**Past falls = 1**	**Past falls = 2**
Non-fallers (*n* = 61)	48	9	4
Fallers (*n* = 31)	16	13	2

### 6-Month Fall Prediction

#### Data Preprocessing

Older adults with missing data were excluded. All assessments were standardized (center to the mean and component-wise scale to unit variance). Multi-collinearity was determined using the Pearson correlation coefficient for the assessments. None of the included predictor variables had a Pearson correlation > 0.7.

#### Classification Experiments

We performed classification experiments to predict 6-month fall risk to see the performance of the classification models. We explored several supervised classification techniques including logistic regression, decision tree, k-nearest neighbors (k-NN), support vector machine classifier (SVM), and random forest for the classification task. The experimental dataset was imbalanced with twice as non-fallers as fallers, a data imbalance often observed in other fall risk prediction studies and consistent with literature findings on the proportion of fallers in a population (2, 9, 10). To address the imbalanced data issue, we used class weights during machine learning model development. For our experiments, we have used the scikit-learn Python library. All of our classification models used a “*balanced”* class weight to address this issue, except for k-nearest neighbors. The “*balanced”* mode uses the values of *samples per class* to automatically adjust weights inversely proportional to class frequencies in the input data (25). The idea of a balanced class weights heuristic is obtained from the article “Logistic regression in rare events data” by King et al. (26, 27).

We performed a hyperparameter grid search for the classifiers to find optimal parameters for the classification task. The hyperparameter grid search was performed to optimize the model parameters for maximum recall. More information on hyperparameter tuning is provided in Supplementary Material 1. We performed five-fold cross-validation for the classification experiments. Results reported are the mean of the five-fold cross-validation performance measures obtained by repeating the classification task 20 times. In addition, for each repetition, we performed random data shuffling before the five-fold cross-validation data splits. We applied regularization techniques, specifically L2 regularization for Logistic regression and an optimized C regularization parameter (a squared L2 penalty) for the SVM classifier to improve overfit in these classifiers. The optimal C parameter was obtained through the hyperparameter grid search of parameters. The classifiers were evaluated based on Area Under the Curve (AUC), validation accuracy (Acc), sensitivity (Sn), specificity (Sp), and F1 scores. The evaluation matrices can be defined as:


$$\begin{array}{lll} Sensitivity\;\left(Sn\right)\; & = & \;\frac{TP}{TP\;+\;FN}\\ Specificity\;\left(Sp\right)\; & = & \;\frac{TN}{TN\;+\;FP}\\ Accuracy\;\left(Acc\right)\; & = & \;\frac{TP\;+\;TN}{TP\;+\;TN\;+\;FP\;+\;FN}\\ \;F1 & = & \;\frac{2TP}{2TP\;+\;FP\;+\;FN} \end{array}$$

Where TP denotes Ture Positive, a test result that correctly indicates the presence of a future fall; TN denotes True Negative, a test result that correctly indicates the absence of a future fall; FP denotes False Positive, a test result which wrongly indicates that a future fall is present; FN denotes False Negative, a test result which wrongly indicates that a future fall is absent; and F1 is the harmonic mean of the precision and recall (28).

We additionally performed calibration tests for the classifiers using Brier score loss to compare the calibration performance of the classifiers (29). We used Python libraries available from scikit-learn to perform the classification analysis (30).

#### Explaining the Models and Individual Predictions Using SHAP

**Figure 1:**
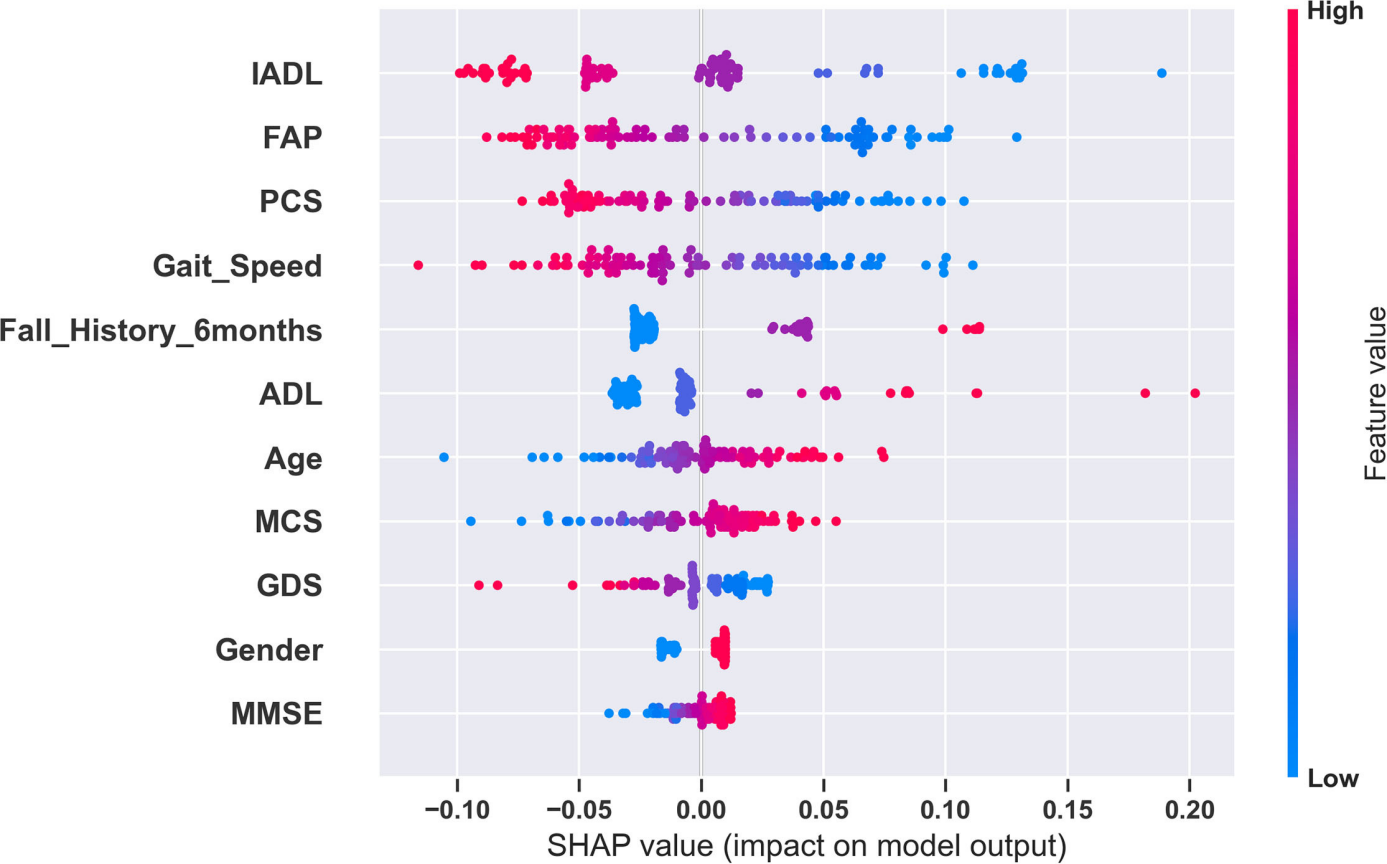
Global explanation of the fall risk model using SHAP. This summary plot shows the relative impact of all predictors over the entire dataset. Each point represents a Shapley value for a predictor and an instance. The colors represent the higher and lower values of the predictor. The features are ordered according to the sum of SHAP value magnitudes over all samples. In this plot, based on the SHAP values for the SVM model, IADL has the highest impact and MMSE has the lowest impact in fall risk prediction.

**Figure 2:**
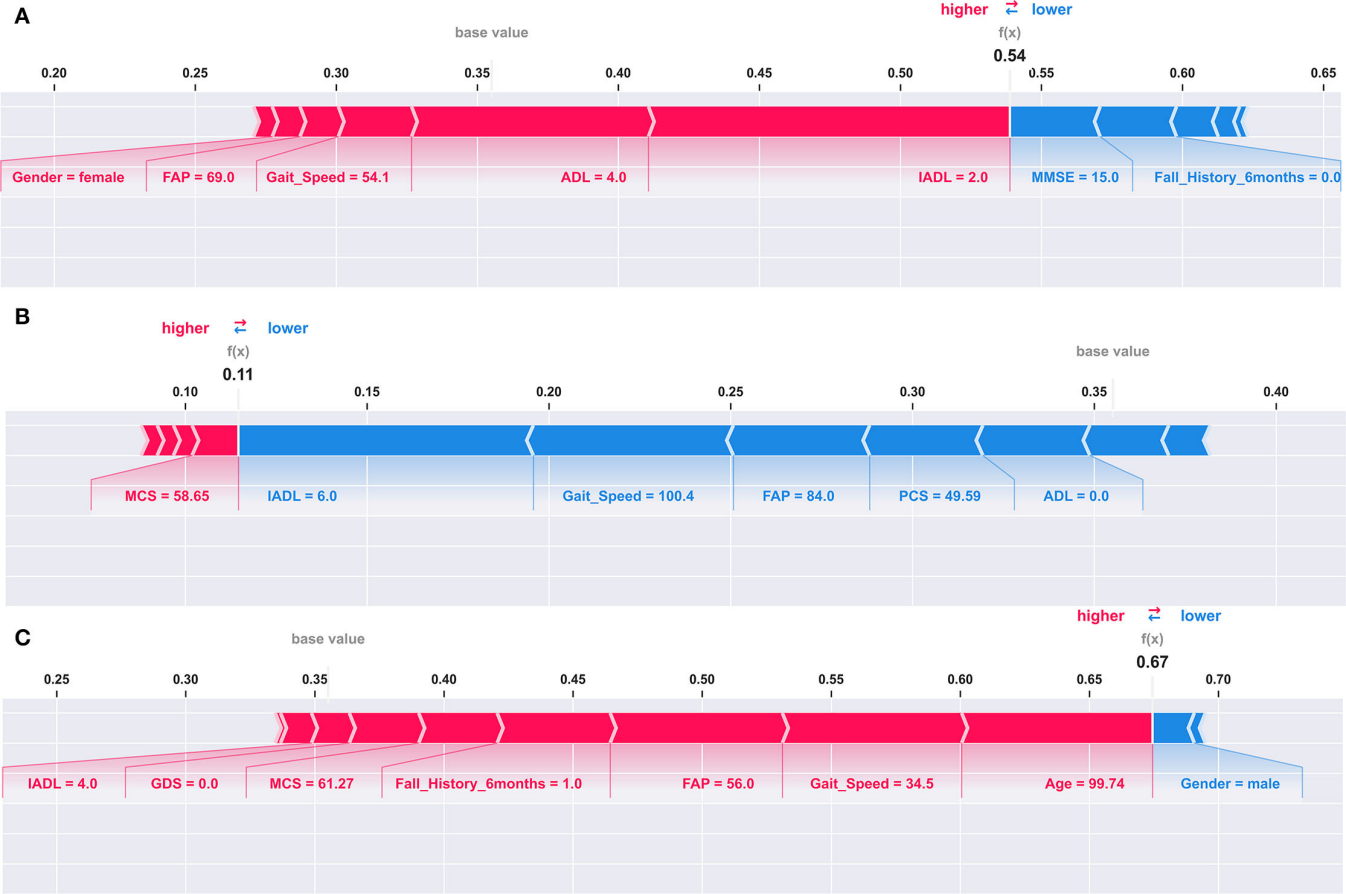
Explaining an individual model prediction for three different TigerPlace residents. The explanation in these plots shows predictors each contributing to push the model output from the base value (the average model output over the training dataset we passed) to the model output for an individual resident. Predictors pushing the prediction higher are shown in red, those pushing the prediction lower are in blue. **(A)** Model prediction explanation for resident 1. **(B)** Model prediction explanation for resident 2. **(C)** Model prediction explanation for resident 3.

Shapley Additive exPlanations (SHAP) values were used to explain our best model and individual predictions (20). SHAP assigns an importance value to each feature for a particular prediction. SHAP values are additive. SHAP values for each feature provide an explanation about which features contributed to either increase or decrease the expected model output. We have included examples in the results section to illustrate how SHAP values show which parameters contributed the most to an individual's high fall risk prediction (20, 31). We used the “shap” Python library developed by Lundberg et al. to perform our analysis (32). For the model explanation, we used the Kernel Explainer methodology implemented in the shap library (20, 32). We used the summary plot feature to plot the summary plot for the SVM classifier in Figure 1 and the force plot feature to plot the individual prediction explanations in Figure 2.

## Results

Table 4 shows the five-fold cross-validation performance measures of the different classifiers predicting 6-month fall risk. Overall, SVM classifier with a linear kernel performed the best with an AUC of 0.80 (95% CI of 0.76–0.85), sensitivity of 0.82 (95% CI of 0.74–0.89), specificity of 0.72 (95% CI of 0.67–0.76), F1 score of 0.76 (95% CI of 0.72–0.79), and accuracy of 0.75 (95% CI of 0.72–0.79). We observed that the decision trees classifier performed with a high sensitivity score of 0.82, similar to SVM. However, it did not perform well in other performance measures. Also, the k-NN classifier performed with the highest specificity of 0.82. However, k-NN did not perform well in other performance measures. In the calibration experiments, we observed that logistic regression, k-nearest neighbors, SVM, and random forest performed similar with Brier score loss values of 0.15, 0.14, 0.15, and 0.14, respectively. The performance of the Decision Tree classifier was poorer with a Brier score loss value of 0.21. Brier score loss is a loss measurement. Therefore, a lower loss value represents more accurate probabilistic predictions. We observed that SVM consistently performed superior across the performance measures of our interest.

**Table 4:** Classification results in predicting 6-month fall risk.

**Classifier**	**Sn (95% CI)**	**Sp (95% CI)**	**F1 (95% CI)**	**Acc (95% CI)**	**AUC (95% CI)**
Logistic regression	0.70 (0.61–0.79)	0.69 (0.64–0.74)	0.70 (0.67–0.73)	0.69 (0.66–0.72)	0.77 (0.71–0.84)
Decision tree classifier	**0.82** (0.70–0.95)	0.45 (0.40–0.50)	0.58 (0.51–0.64)	0.57 (0.52–0.63)	0.63 (0.56–0.71)
k-NN	0.53 (0.39–0.68)	**0.82** (0.75–0.89)	0.71 (0.64–0.78)	0.72 (0.65–0.78)	0.78 (0.73–0.82)
SVM (kernel = linear)	**0.82** (0.74–0.89)	0.72 (0.67–0.76)	**0.76** (0.72–0.79)	**0.75** (0.72–0.79)	**0.80** (0.76–0.85)
Random forest	0.74 (0.64–0.84)	0.72 (0.68–0.77)	0.73 (0.69–0.78)	0.73 (0.69–0.77)	0.78 (0.74–0.83)

*Bold indicates best result in the corresponding column*.

### Explaining the Models and Individual Predictions Using SHAP

We used SHAP to explain our best model and model predictions. Figure 1 shows the global explanation of the SVM model with a linear kernel. The plot shows how the feature importance values are distributed for each feature. For example, lower values of gait speed correspond to higher SHAP values and vice versa. Therefore, during a model prediction, a lower value of gait speed would increase the fall risk prediction. Figures 2A–C provide explanations of falls risk predictions for three different older adults. Explanations of individual model predictions provide details about which predictor variables contributed to increase or decrease the fall risk for an older adult.

Figure 2A shows that a lower IADL of 2 and an increased ADL of 4 were the largest contributors to elevate the fall risk for Resident 1. A lower gait speed of 54.1 and a lower FAP score of 69 also contributed to increase the fall risk for Resident 1. However, IADL had the largest contribution, followed by ADL for the elevated fall risk. In addition, an average MMSE of 15 and not having a history of falls contributed to reduce the fall risk for Resident 1. The fall risk prediction presented in Figure 2B shows that a higher IADL of 6, a higher gait speed of 100.4, and a higher FAP of 84 were the three largest contributors to reduce the fall risk for Resident 2. In our third example of individual fall risk prediction shown in Figure 2C, we observed that a higher age of about 100, a lower gait speed of 34.5, a lower FAP score of 56, including a history of previous falls contributed to an elevated the fall risk for Resident 3. The SVM model used for the SHAP explanations had an expected base fall prediction value of 0.36.

## Discussion

We observed that the SVM model with a linear kernel performed superior to other models in the classification task of predicting 6-month fall risk. Explanations to the model predictions using SHAP values provide additional insights about which predictor variables have contributed to increase or decrease fall risk prediction for an individual. Understanding which predictor variables are contributing to elevate the fall risk may help health providers to provide personalized interventions to the residents. Therefore, the fall risk model could provide essential guidance to a health provider to focus on specific factors of fall risk instead of analyzing the individual assessments or predictor variables to understand their effects. For example, in Figure 2A we observed that the fall risk for the individual was predicted to be 0.54 suggesting the resident had a relatively higher fall risk as compared to the base fall risk of 0.36. The SHAP explanation to the individual prediction shows that the ADL, IADL assessment scores, and gait speed were the three most prominent contributors to the relatively higher fall risk. Intuitively, we can also observe that having no fall history is helping Resident 1 to reduce the fall risk. Similarly, in Figure 2B we can observe that the fall risk predicted by the model is 0.11, indicating the resident had a lower fall risk at the time of assessments. Evaluation of the SHAP values for this individual prediction suggests that a significantly higher gait speed and IADL contributed to reduce the fall risk for the resident. In Figure 2C, we observed that the resident had a higher fall risk of 0.67. Evaluation of SHAP values for the resident suggests that the key contributors for higher fall risk were age, gait speed, and FAP. These critical and objective explanations could help clinicians save time and provide focused interventions to older adult residents with increased fall risk.

Analyzing the SVM classifier coefficients and SHAP values associated with the predictor variables, we observed that predictor variables related to physical function and performance had a greater influence in predicting future falls. The predictor variables with the most influence in fall risk prediction were ADL, IADL, FAP, Gait Speed, and PCS. We also observed that gender did not play a significant role in predicting fall risk.

The dataset used in this study is imbalanced. In the dataset, we observed twice as many non-fallers as compared to fallers. We have extensively evaluated the models to reduce model overfit. A larger dataset with more participants could improve the generalizability of the model.

In addition, future analysis with more predictor variables could potentially improve the results. Grip Strength, TUG scores, and medications could be included to improve the prediction. Also, a longitudinal study with repeated measures from the individual subjects for recurrent events (fall and hospitalization) could provide improved and personalized risk assessment scores.

The model developed in this study is built using limited data. We believe this can be successfully used for fall risk prediction at TigerPlace and in similar populations but cannot be generalized to all older adult populations. The key objective of this study was to develop a multifactorial fall risk prediction system that can provide explanations for individual fall predictions, which can help clinicians to provide personalized care.

## Conclusions

We have performed an analysis to predict 6-month fall risk among older adults using geriatric assessments, spatiotemporal gait parameters, and fall history. Out of the several assessments and spatiotemporal measurements, ADL, IADL, FAP, Gait Speed, and PCS were generally better predictors of 6-month fall risk. The fall risk models could potentially help clinicians to save time from analyzing individual assessments and provide early interventions to avoid a possible future fall. Also, the prediction explanations using SHAP could potentially help the clinicians to provide targeted interventions that are personalized to the individual.

## Data Availability Statement

The original contributions presented in the study are included in the article/Supplementary Material, further inquiries can be directed to the corresponding author.

## Ethics Statement

The studies involving human participants were reviewed and approved by Institutional Review Board, University of Missouri, Columbia. The patients/participants provided their written informed consent to participate in this study.

## Author Contributions

AKM conducted the cleaning, organizing, analysis of data including writing software programs to de-identify EMR data, developed the fall risk prediction model and performed the statistical analysis, and drafted the manuscript. SM collected the fall events data and provided feedback on de-identifying EMR data. MS, LD, MP, JK, MR, CA, and AKM participated in defining the research questions, context, and purpose of the study. MS, LD, MP, JK, MR, CA, ME, and SS provided counseling and feedback on the study methodology. All authors have read, reviewed, and approved the manuscript.

## Funding

This work was supported in part by the National Institute of Nursing Research of the National Institutes of Health under award number R01NR016423 (Skubic, PI).

## Author Disclaimer

The content is solely the responsibility of the authors and does not necessarily represent the official views of the NIH.

## Conflict of Interest

The authors declare that the research was conducted in the absence of any commercial or financial relationships that could be construed as a potential conflict of interest.

## Publisher's Note

All claims expressed in this article are solely those of the authors and do not necessarily represent those of their affiliated organizations, or those of the publisher, the editors and the reviewers. Any product that may be evaluated in this article, or claim that may be made by its manufacturer, is not guaranteed or endorsed by the publisher.
