# Supplementary material for: Explainable Fall Risk Prediction in Older Adults Using Gait and Geriatric Assessments
Source: Front Digit Health. 2022 May 6;4:869812. doi: 10.3389/fdgth.2022.869812 (PMC9120414; doi:10.3389/fdgth.2022.869812)
Supplement: Supplementary file 1 [file Data_Sheet_1.DOCX]

**Supporting Document - 1**

**Explainable Fall Risk Prediction in Older Adults Using Gait and Geriatric Assessments**

**Hyperparameter Search for Classifiers**

Hyperparameter tuning for each model was conducted using *GridSearchCV* model selection technique available in *scikit-learn* Python Library (1). Please find the list of machine learning models, their grid search parameters, and the final selected parameters for maximum recall. We used the model implementations available in scikit-learn.

1. Logistic regression (model implementation: sklearn.linear_model.LogisticRegression):

Logistic regression does not depend on several parameters when compared with other classifiers. We used ‘liblinear’ solver because of the smaller size of our dataset. We also used L2 regularization and ‘balanced’ class weight for this classifier. We did not perform a hyperparameter search for this model. This is our baseline model.

1. Decision Tree (model implementation: sklearn.tree.DecisionTreeClassifier):

Grid Search Parameters:

criterion = ['gini', 'entropy']

splitter = ['best', 'random']

max_depth = [2, 3, 4, 5, 6, 7, 8, 9, 10, 11, 12, 13, 14, 15]

min_samples_split = [2, 3, 4, 5, 7, 8, 9, 10, 11, 12, 13]

min_samples_leaf = [ 1, 3, 4, 5, 6, 7, 8, 9, 10]

min_weight_fraction_leaf = [0.0]

max_features = ['auto', 'sqrt', 'log2']

random_state = [42]

class_weight = [None, 'balanced']

Selected Parameters:

criterion='gini'

max_depth=2

max_features='auto'

min_samples_leaf=5

min_samples_split=2

min_weight_fraction_leaf= 0.0

random_state= 42

splitter='random'

class_weight='balanced'

1. KNN (model implementation: sklearn.neighbors.KNeighborsClassifier):

Grid Search Parameters:

n_neighbors = [2, 3, 4, 5, 6, 7, 8, 9, 10, 11, 12]

weights = ['uniform', 'distance']

algorithm = ['auto', 'ball_tree', 'kd_tree', 'brute']

p=[1, 2]

Selected Parameters:

algorithm='auto'

n_neighbors=7

p=1

weights='uniform'

1. SVM (model implementation: sklearn.svm.SVC):

Grid Search Parameters:

C = [0.001, 0.01, 0.1, 1.0, 1.5, 2.0, 2.5]

kernel = ['linear', 'poly']

gamma = ['auto', 'scale']

class_weight = [None, 'balanced']

Selected Parameters:

kernel='linear'

gamma='auto'

C=0.01

class_weight='balanced'

1. Random Forest (model implementation: sklearn.ensemble.RandomForestClassifier):

Grid Search Parameters:

n_estimators = [20, 30, 50, 100]

criterion = ['gini', 'entropy']

max_depth = [2, 3, 4, 5, 6, 7, 8]

min_samples_split = [2, 3, 4, 5]

min_samples_leaf = [ 1, 3, 4, 5, 6, 7, 8, 9, 10]

max_features = ['auto']

random_state = [42]

class_weight = ['balanced', 'balanced_subsample']

Selected Parameters:

n_estimators = 50

criterion = 'gini'

max_depth = 3

min_samples_split = 2

min_samples_leaf = 9

max_features = 'auto'

andom_state = 42

class_weight = 'balanced_subsample'

References:

1. Pedregosa F, Varoquaux G, Gramfort A, Michel V, Thirion B, Grisel O, et al. Scikit-Learn: Machine Learning in Python. *the Journal of machine Learning research* (2011) 12:2825-30.
